# Bacteria-Mediated RNA Interference for Management of *Plagiodera versicolora* (Coleoptera: Chrysomelidae)

**DOI:** 10.3390/insects10120415

**Published:** 2019-11-21

**Authors:** Yiqiu Zhang, Letian Xu, Shengchun Li, Jiang Zhang

**Affiliations:** State Key Laboratory of Biocatalysis and Enzyme Engineering, Hubei Collaborative Innovation Center for Green Transformation of Bio-Resources, School of Life Sciences, Hubei University, Wuhan 430062, China; 201711110710953@stu.hubu.edu.cn (Y.Z.); letian0926@163.com (L.X.)

**Keywords:** *Plagiodera versicolora*, RNA interference, double-stranded RNA, bacteria-expressed dsRNA, target genes

## Abstract

RNA interference (RNAi) has emerged as a novel and feasible strategy for pest management. Methods for cost-effective production and stable delivery of double-stranded RNA (dsRNA) to the target insects are crucial for the wide application of RNAi for pest control. In this study, we tested the expression of dsRNA in RNaseIII-deficient *Escherichia coli* HT115 which was then fed to *Plagiodera versicolora* larvae, an insect pest of Salicaceae plants worldwide. By targeting six potential genes, including actin (*ACT*), signal recognition particle protein 54k (*SRP54*), heat shock protein 70 (*HSC70*), shibire (*SHI*), cactus (*CACT*), and soluble N-ethylmaleimide-sensitive fusion attachment proteins (*SNAP*), we found that feeding bacteria-expressed dsRNA successfully triggered the silencing of the five target genes tested and the suppression of *ACT* and *SRP54* genes caused significant mortality. Our results suggest that the oral delivery of bacteria-expressed dsRNA is a potential alternative for the control of *P. versicolora*, and that *ACT* and *SRP54* genes are the potent targets.

## 1. Introduction

*Plagiodera versicolora* (Coleoptera: Chrysomelidae), one of the most important pests of Salicaceae plants, is widely distributed across Asia, Europe, and northern Africa [1,2]. Both larvae and adults of the *P. versicolora* feed on the leaves of willow and poplar, leaving only the midrib and a network of veins. Although chemical insecticides can efficiently control this pest, their long-term application inevitably leads to undesirable and hazardous side effects to the environment, plants, and human [3]. *Bacillus thuringiensis* (Bt) toxin engineered to be expressed in plants is another method for *P. versicolora* control [4,5,6], but it would potentially encounter the problem of evolution of insect resistance [7,8].

RNA interference (RNAi) is a highly conserved post-transcriptional regulation mechanism in eukaryotes that is initiated by the presence of double-stranded RNA (dsRNA) [9,10]. It was first reported in the nematode, *Caenorhabditis elegans* [11]. Since then, RNAi technology has been utilized as a powerful reverse genetics tool for studying gene function in insects by suppression of gene expression [12,13], and it has also shown great potential in pest management [14,15]. When dsRNAs whose sequences are derived from essential insect genes are delivered into insect cells, silencing of the target genes through induction of RNAi could be triggered, a process also dubbed as environmental RNAi. It was demonstrated that insects feeding on transgenic plants producing dsRNAs targeted against essential insect genes resulted in significant suppression of the target gene, which led to increased mortality of *Diabrotica virgifera virgifera* and *Helicoverpa armigera* [16,17].

There are several factors which may be responsible for the efficacy of RNAi responses in insects, such as target gene, administered dose of dsRNA used [18], and delivery methods as well [19]. Identification of optimal target genes is a key step to achieve pest control with RNAi, while the screening of many target genes requires an efficient dsRNA delivery. Recently, Ulrich et al. [20] identified 11 highly efficient RNAi target genes for controlling the red flour beetle *Tribolium castaneum*, showing 100% larval lethality eight days postinjection of dsRNA. In addition, five *D. v. virgifera* RNAi target orthologs from Baum et al. [17] were selected for testing in *T. castaneum* also showed high degree of mortality, indicating that RNAi target genes may be leveraged across Coleoptera [20]. Thus, we hypothesized that these *T. castaneum* target genes might also be effective in *P. versicolora*. In this study, we selected five *T. castaneum* targets, including cactus (CACT, a part of the Toll signaling pathway) [21], heat shock protein 70 (HSP70), signal recognition particle protein 54k (SRP54, a key component of the ribonucleoprotein complex that mediates the co-translational targeting of secretory and membrane proteins to the endoplasmic reticulum) [22], soluble N-ethylmaleimide-sensitive fusion attachment proteins (SNAP, involved in intracellular membrane fusion and vesicular trafficking) [23], and shibire (SHI, a protein with GTPase activity required for endocytosis) [24]. In addition, *β*-actin (ACT), a sensitive RNAi target of *Leptinotarsa decemlineata*, was also selected [25].

The basic dsRNA delivery methods consists of microinjection, feeding, topical application, and soaking [19]. The most common approach for dsRNA delivery is microinjection, in which exact amounts of dsRNA is directly injected into insect hemocoel [26]. However, microinjection is laborious and disruptive, and is sometimes challenging due to the small body size of the insects. Topical application and soaking approaches would encounter structural barriers of insects, such as the integument, which could prevent penetration of dsRNA delivery [27]. Feeding is non-invasive and eliminates mechanical injury to the insect. In comparison with producing dsRNA in vitro with a kit, the application of microorganism-expressed dsRNA is cost-effective, and is easy for large scale gene function analysis and practical application in the field. Oral delivery of bacterially expressed dsRNA has been reported to be highly effective for RNAi in some Coleopteran insects [17,28,29,30], which might also be a potential approach to control *P. versicolora*, a pest of forestry. In this study, using green florescent protein (*GFP*) as a control, we cloned the fragments from *GFP* and six target genes into L4440 plasmid, a recombinant expression vectors comprising two convergent T7 promoters, and then transformed them into an RNaseIII-deficient *Escherichia coli* strain HT115. By feeding dsRNA-expressing bacteria as an oral delivery method to assess its insecticidal effect to *P. versicolora* larvae, we found that *ACT* and *SRP54* genes caused the highest mortality.

## 2. Material and Methods

### 2.1. *Plagiodera Versicolora* (Laicharting) Rearing

The *P. versicolora* adults were collected from willow at Sha Lake Park in Wuhan (30.35° N, 114.33 E), Hubei Province in China. Newly-hatched larvae were reared using fresh willow leaves in an insectary with the condition of at 25 ± 2 °C, 50–60% relative humidity, and a photoperiod of 14 h light/10 h dark.

### 2.2. *Plagiodera Versicolora* RNA Extraction and Quantitative PCR (qPCR) Analysis

The total RNA was isolated from insect samples using RNAiso Plus Reagent (Takara, Dalian, China) according to the manufacturer’s instructions. Hifair^®^ II 1st Strand cDNA Synthesis Kit with gDNA digester (Yeasen, China) was used to remove genomic DNA and synthesize cDNA. The levels of mRNA expression were quantified by qPCR with SYBR^®^ Premix Ex Taq™ II (Tli RNaseH Plus) (Takara, Dalian, China) on Bio-Rad CFX Connect Real-Time System (Bio-Rad, USA). The reaction was performed in a final volume of 10 µL containing 2 × SYBR^®^ Premix Ex Taq™ II, 2 µL of cDNA and 0.25 µL of each primer (Table S1, 10 µM). The reaction conditions were as follow: 95 °C for 2 min, followed by 40 cycles at 95 °C for 5 s, and then 60 °C for 30 s. The 40S ribosomal protein S18 (*RPS18*) gene was used as an internal control to normalize for specific gene expression in the samples. Each treatment was replicated with three independent biological sample preparations. Quantitative analysis of gene expression was performed using the 2^−ΔΔCT^ method [31].

### 2.3. Construction of Bacterial Expression Vectors for dsRNA Production

**Figure 1:**
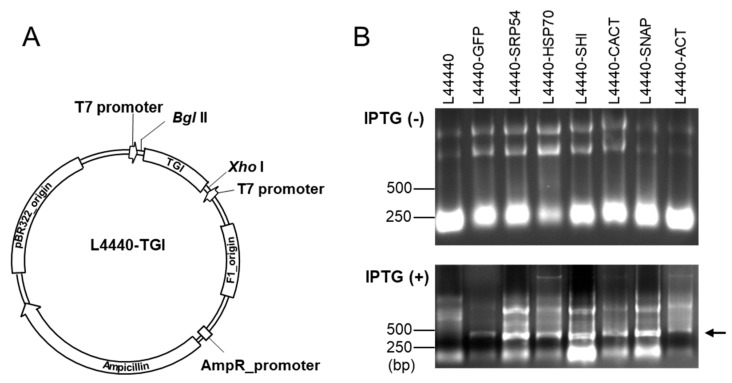
Schematic diagram of the *E. coli-*expressed dsRNA production system. (**A**) The target gene of interest (TGI) was inserted into the expression vector (L4440) through homologous recombination [32,33]. (**B**) Production of dsRNA with an inducer, IPTG. Transformed *E. coli* with L4440-TGI expressed dsRNA in the presence of IPTG. Arrow indicates the dsRNA production upon IPTG induction.

To construct a recombinant plasmid to express dsRNA, the L4440 vector (plasmid Addgene 1654) was digested with *Bgl* II and *Xho* I (Figure 1A). Then, the target-gene fragment was amplified from the larval cDNA with specific primer pairs (Table S1) and cloned into L4440 vector via a homologous recombination [32,33]. The mixtures were transformed into competent *E. coli* DH5α cells. Positive colonies were confirmed by a DNA sequencing before transformation into *E. coli* HT115.

### 2.4. Expression of dsRNA and Larval Feeding Bioassay

The *E. coli* HT115 containing the recombinant L4440 with *GFP* or target gene was cultured in Luria–Bertani (LB) medium with ampicillin (100 μg/mL) and tetracycline (12.5 μg/mL) at 37 °C with shaking at 120 rpm overnight. Then, the culture was diluted 100× with fresh LB medium with the same antibiotics as above and incubated at 37 °C at 120 rpm until OD_600 nm_ reached 0.4. To activate the T7 promoter for RNA transcription, 0.4 mM (final concentration) isopropyl *β*-d-1-thiogalactopyranoside (IPTG) was added and then incubated for an additional 5 h at the same conditions. Presence of the synthesized dsRNA was evaluated using 1% agarose gel electrophoresis.

After induced overexpression of dsRNA, the cultured broth was centrifuged at 5000× *g* for 2 min and bacterial pellet was resuspended in autoclaved ddH_2_O until OD_600 nm_ attained 1.0. For bioassay to assess the insecticidal activity of dsRNA, 50 μL of bacterial suspensions were painted on fresh willow leaves (4 cm^2^) and fed to first instar larvae of *P. versicolora* (N = 40). ddH_2_O-painted leaves were served as control The insect feeding bioassay was performed under controlled conditions at 25 ± 2 °C, 14 h photoperiod and 50–60% relative humidity. The feed was refreshed and mortality rates were tracked daily for 7 d. The whole experiments were repeated four times.

### 2.5. Statistical Analysis

Survival curves were analyzed using the Kaplan–Meier method and the log-rank test was used to evaluate the significance of differences between two groups. qRT-PCR data was analyzed with one-way ANOVA coupled with Bonferroni posterior test. Data were analyzed by SPSS 19.0 (SPSS Inc., Chicago, IL, USA). A value of *P* < 0.05 was considered significantly different.

## 3. Results

### 3.1. Analysis of dsRNA Induced in Bacteria

To produce bacteria-expressed dsRNA, a partial sequence of target gene of interest was inserted between two T7 RNA polymerase promoters of L4440 vector (Figure 1A). The recombinant vector was transformed to *E. coli* HT115 lacking RNase ΙΙΙ. The transformed *E. coli* was induced to overexpress dsRNA by IPTG addition, which was confirmed by electrophoresis on 1% agarose gel (Figure 1B).

### 3.2. Insecticidal Activity of dsRNA-Expressing *E. coli* against *Plagiodera versicolora* Larvae

**Figure 2:**
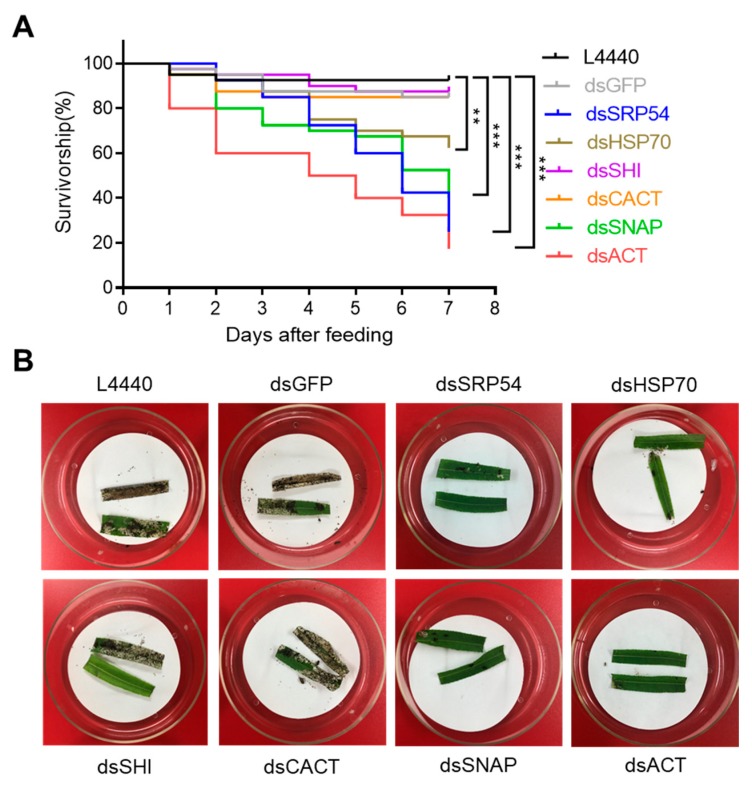
Effects of different dsRNAs expressed in bacteria on the survivorship of the 1st instar larvae. (**A**) Kaplan–Meier survival curves of *P. versicolora* (N = 40) fed with willow leaves painted with indicated dsRNA-expressing *E.coli*. The asterisk denotes significant differences (** *P* < 0.01, *** *P* < 0.001). (**B**) Example of a bioassay with willow leaves painted with indicated dsRNA-expressing *E.coli*. Leaves were fed to first instar *P. versicolora* larvae (N = 40) for four days.

*P. versicolora* larvae fed with fresh willow leaves painted with *E. coli* HT115 expressing dsRNA targeting *ACT*, *HSP70*, *SNAP* and *SRP54* showed a significant increase in mortality compared to all negative controls (ddH_2_O, empty vector L4440 and ds*GFP*, Figure 2 and Figure S1). After four days of dsRNA administration, mortality ranging from 26% to 30% was recorded by feeding dsRNA targeting *HSP70*, *SNAP* and *SRP54*, while providing dsRNA targeting *ACT* exhibited ~50% mortality (Figure 2A). Obvious antifeedant activities were observed for larvae that were fed dsRNA of these four genes (Figure 2B). Among the six genes targeted, feeding of ds*SRP54* and ds*ACT* was most effective in *P. versicolora* larvae, causing the highest mortality (75% and 82% mortality, respectively), following seven days of dsRNA administration (Figure 2A).

### 3.3. Target Gene Silencing of *Plagiodera versicolora* Larvae after Feeding Bacteria-Expressed dsRNA

**Figure 3:**
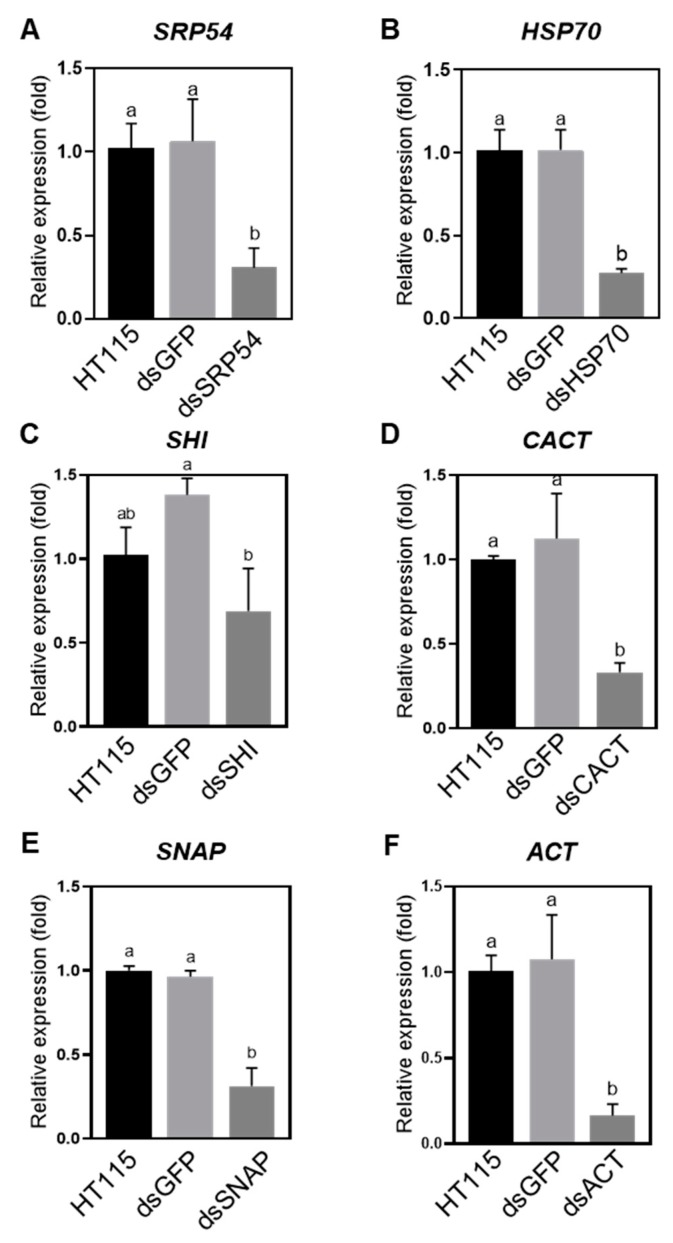
Relative gene expression of *Plagiodera versicolora* larvae (n = 40) after being fed for 3 days with willow leaves painted with bacteria-expressed dsRNAs targeting indicated genes: (**A**) *SRP54*. (**B**) *HSP70*. (**C**) *SHI*. (**D**) *CACT*. (**E**) *SNAP*. (**F**) *ACT*. Relative expression of indicated genes were normalized using *RPS18* as a reference gene. Bars on columns indicate the standard error (SE). Different letters on the columns mean significant difference by the Tukey at a 5% significance level.

To confirm that the killing of the *P. versicolora* larvae by feeding *E. coli* HT115-expressed dsRNA was due to induction of gene silencing, we determined expression of the target genes in the larvae after three days of feeding. We showed that, after three days, except for a slight reduction of *SHI*, five other target genes were silenced successfully (Figure 3). Reduced expression of *CACT* and *SHI* did not cause increased mortality (Figure 2A and Figure 3C,D), which suggests that these two genes may not be indispensable for *P. versicolora*.

## 4. Discussion

RNAi technique has been exploited as a useful tool for pest management [14,15]. *P. versicolora* is an economically important pest posing threat to Salicaceae plants. Our aim was to examine whether there is an RNAi response in *P. versicolora* and also screen optimal target genes for RNAi. In this study, we demonstrated that oral delivery of bacteria-expressed dsRNA can specifically inhibit the expression of target genes in *P. versicolora* (Figure 3). We evaluated six potential genes for use as targets to control *P. versicolora* by RNAi. Silencing the expression of four target genes by administration of bacteria-expressing dsRNA can result in increased mortality in *P. versicolora* compared with control groups (Figure 2A). *ACT*, caused the highest mortality following dsRNA administration (Figure 2A). CACT are supposed to have essential biological function, however, specific suppression of their expression did not cause increased mortality (Figure 2 and Figure 3). Interestingly, it was demonstrated that oral treatment of a combination of ds*SHI* and ds*HSP70* at lower concentration by neonates and adults of *Agrilus planipennis* efficiently elicited mortality. Exposing adults to 1 μg/μL of ds*SHI* plus ds*HSP70* caused 90% mortality after two weeks, whereas, 10 μg/μL of single dsRNA treatment exhibited lower mortality (up to 40%) [34]. Therefore, it may be worthwhile to test whether there is a combined effect by silencing both *SHI* and *HSP70* in *P. versicolora*.

Using bacterial expression systems, production of dsRNA can easily be scaled-up and remains cost-effective, and these bacteria could be sprayed directly on crops at any time. Thus, the evaluation of the bacterial delivery system described in this study, apart from serving as an efficient system to screen candidate gene targets in the laboratory, provides an avenue to control *P. versicolora* utilizing dsRNA under field conditions. Nevertheless, there are several knowledge gaps that need to be fulfilled before bacteria-expressed dsRNA can be used in pest management. Concerns associated with potential effects on off-target, nontarget organisms and potential resistance development, such as mutations of target genes, mutations of RNAi core machinery genes, and enhanced dsRNA degradation [19], need to be taken into consideration. In addition, as the transgenic *E. coli* is not part of insect microbial flora, it might induce an immune response [35,36]. Therefore, isolation and engineering of symbiont bacteria for dsRNA production and delivery is also a perspective for pest control [37,38,39].

The production of transgenic plants is another strategy to deliver dsRNA for inset pest control. A number of studies have used this approach to develop transgenic plants expressing dsRNA against insects in the order Hemiptera [40,41,42], Coleoptera [17,43,44,45], or Lepidoptera [16,46,47]. However, transgenic plant-mediated RNAi based on conventional nuclear transformation technology did not offer efficient protection from insect damage, probably due to (i) low expression of dsRNA and (ii) instability of dsRNAs (result from plants’ endogenous Dicer cleavage) in planta [14]. However, the use of genetically modified organisms has raised considerable scientific and public concerns, such as gene flow. Producing dsRNA in a plant’s plastids (chloroplasts) lacking RNAi machinery, rather than its cellular cytoplasm, would be a preferable strategy to minimize gene flow and achieve improved results [25,48]. Most recently, we have developed an efficient and stable plastid transformation protocol for poplar [49], future work is needed to investigate whether transplastomic poplar expressing dsRNA targeting *ACT* or/and *SRP54*, the two most potent targets identified in this study, are resistant to *P. versicolora.* Also, it would be worth simultaneously expressing dsRNA and Bt toxin from the poplar genome to control *P. versicolora* [50].

## 5. Conclusions

In this study, our results indicated that ingestion of bacteria-expressed dsRNA could induce RNAi response in *P. versicolora,* and *SRP54* and *ACT* represent candidate genes for RNAi-based control of *P. versicolora*. Our results also threw light on the application of bacteria-expressed dsRNA for controlling *P. versicolora*.

## Supplementary Materials

The following are available online at https://www.mdpi.com/2075-4450/10/12/415/s1, Figure S1: No effects of dsGFP-expressing bacteria on the survivorship of the 1st instar larvae. Table S1: Oligonucleotide sequences used in this study (homologous sequences are underlined).
