# Supplementary material for: Bacteria-Mediated RNA Interference for Management of Plagiodera versicolora (Coleoptera: Chrysomelidae)
Source: Insects. 2019 Nov 21;10(12):415. doi: 10.3390/insects10120415 (PMC6955681; doi:10.3390/insects10120415)
Supplement: Supplementary file 1 [file insects-10-00415-s001.pdf]

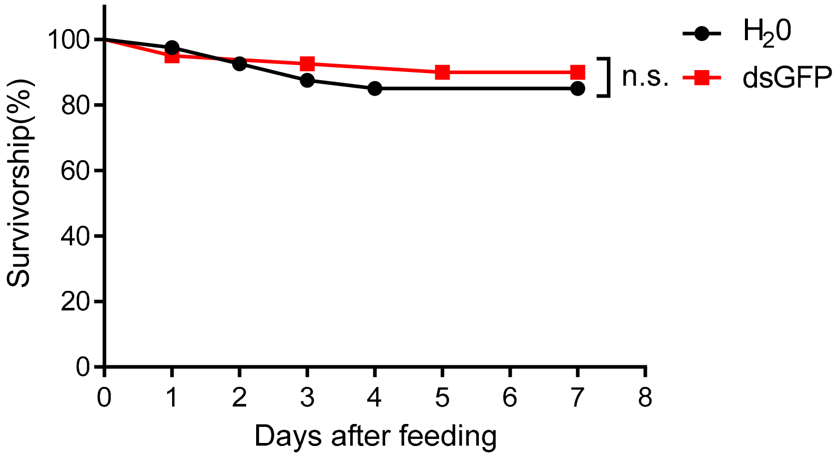

Figure 1. No effects of ds*GFP*-expressing bacteria on the survivorship of the 1st instar larvae. Not significant (n.s.) difference between the survival of H_2O-treated and dsGFP-treated *P. versicolora* larvae.

Table S1. Oligonucleotide sequences used in this study (homologous sequences are underlined).

Primer	Sequence (5'-3')	Amplicon (bp)
F-dsRNA-GFP	ACTATAGGGAGACCGGCAGATCTGAAGTGGAGAGGGTGAAGGTGAT	381
R-dsRNA-GFP	GGTACCGGGCCCCCCCTCGAGGTCGCTTGTCGGCCATGATGTATAC	
F-dsRNA-ACT	ACTATAGGGAGACCGGCAGATCTGAACCCCTGCCATGTACGTCGCT	368
R-dsRNA-ACT	GGTACCGGGCCCCCCCTCGAGGTCGCCGATGGTGATGACTTGTCCG	
F-dsRNA-CACT	ACTATAGGGAGACCGGCAGATCTGACCAACTTCCTCGACATGCACAA	346
R-dsRNA-CACT	GGTACCGGGCCCCCCCTCGAGGTCGACCAACTGTCTGAGAACGTCGA	
F-dsRNA-HSP70	ACTATAGGGAGACCGGCAGATCTGAGCTCTCACACTCGCGCGCACTT	335
R-dsRNA-HSP70	GGTACCGGGCCCCCCCTCGAGGTCGTGAAGAACCAGCTCGGCGACAA	
F-dsRNA-SHI	ACTATAGGGAGACCGGCAGATCTGACCCGCAGCTCAAGAGGCAGGT	351
R-dsRNA-SHI	GGTACCGGGCCCCCCCTCGAGGTCGACCGCTGTAGATTTTCCACCAC	
F-dsRNA-SNAP	ACTATAGGGAGACCGGCAGATCTGATTGAGAAGGCATTTGTTAGCTGAA	373
R-dsRNA-SNAP	GGTACCGGGCCCCCCCTCGAGGTCGAATGTTACCAAAGAGCAGCAAATC	
F-dsRNA-SRP54	ACTATAGGGAGACCGGCAGATCTGAATGTGTTCACCTGTGCCTATG	340
R-dsRNA-RP54	GGTACCGGGCCCCCCCTCGAGGTCGTGGATCCTGTGGTTATCGCT	
F-qPCR-ACT	CGTGACTTGACCGACTACCT	118
R-qPCR-ACT	CGAGAGCGACATAGCAGAGT	
F-qPCR-CACT	CAGCTATCAAGGTCATTCG	117
R-qPCR-CACT	ACCAACTGTCTGAGAACG	
F-qPCR-HSC70	CTGGTACAACTTGGCGATA	101
R-qPCR-HSC70	AGTGGCTGGAGGATAACC	
F-qPCR-RPS18	CTTCCTCGTCGGAGCATTCT	110
R-qPCR-RPS18	GTTCGCCTTAACTGCCATCAA	
F-qPCR-SHI	TCAAGAGGCAGGTGGAGAC	87
R-qPCR-SHI	TCTTCGGCACCAAGTCCTT	
F-qPCR-SNAP	AACAGTTCACTGCCTCATTGAT	86
R-qPCR-SNAP	TGGTGCCAGACATGATGCT	
F-qPCR-SRP54	ACTCCTCCTGCTTATGTCTACC	98
R-qPCR-SRP54	TCCTGTGGTTATCGCTCAAGA	
